# Risk of bone metastases on [^18^F]NaF PET/CT in 734 patients with newly diagnosed prostate cancer

**DOI:** 10.1007/s00259-026-08148-y

**Published:** 2026-09-05

**Authors:** Claus Madsen, Helle D. Zacho, Rasmus Bisbjerg, Dan Fuglø, Peter B. Østergren

**Affiliations:** 1 Department of Nuclear Medicine, Copenhagen University Hospital Herlev and Gentofte, Borgmester Ib Juuls Vej 31, 2730 Herlev, Denmark; 2 Department of Nuclear Medicine and Clinical Cancer Research Centre, Aalborg University Hospital, Hobrovej 18-22, 9000 Aalborg, Denmark; 3 Department of Clinical Medicine, Aalborg University, Aalborg, Denmark; 4 Department of Urology, Copenhagen University Hospital Herlev and Gentofte, Borgmester Ib Juuls Vej 1, 2730 Herlev, Denmark; 5 Department of Clinical Medicine, University of Copenhagen, Copenhagen, Denmark

**Keywords:** Prostate cancer, Bone metastases, NaF PET/CT, Intermediate-risk, Diagnostic accuracy

## Abstract

**Purpose:**

International guidelines recommend assessment for bone metastases in patients with high-risk prostate cancer (PC) and those with unfavourable intermediate-risk disease. These recommendations are largely based on studies using planar bone scintigraphy. We aimed to assess the diagnostic accuracy of [^18^F]sodium fluoride ([^18^F]NaF) PET/CT in newly diagnosed PC. We also evaluated the clinical utility of routine bone imaging in patients with unfavourable intermediate-risk disease and International Society of Urological Pathology grade group (ISUP GG) 3.

**Methods:**

This retrospective single-center study included 734 consecutive patients with newly diagnosed PC who underwent [^18^F]NaF PET/CT for primary staging. Patients were classified according to the current European Association of Urology risk classification, with the intermediate-risk subgroup restricted to patients with ISUP GG 3. All scan reports were retrospectively reviewed, and patient-level findings regarding bone metastases were validated against a reference standard based on follow-up imaging and clinical data. Sensitivity, specificity, and predictive values were calculated.

**Results:**

Of the 734 patients, 607 (83%) had high-risk disease and 127 (17%) had intermediate-risk disease with ISUP GG 3. Overall, bone metastases were detected in 225 patients (31%). Among high-risk patients, 224 (37%) had bone metastases, whereas only 1 of 127 intermediate-risk patients (0.8%) had bone metastases on [^18^F]NaF PET/CT. Follow-up identified one additional potential false-negative case in the intermediate-risk group. Among patients with a conclusive reference standard (*n* = 689), sensitivity and specificity were 0.90 and 0.99, respectively.

**Conclusion:**

[^18^F]NaF PET/CT demonstrates high diagnostic accuracy for the detection of bone metastases in newly diagnosed PC. However, given the very low prevalence of bone metastases, its clinical utility in patients with unfavourable intermediate-risk disease and ISUP GG 3 appears limited.

## Introduction

Prostate cancer (PC) frequently metastasises to the bones and lymph nodes. Prostate-specific antigen (PSA) level, clinical tumour stage (T-stage), and International Society of Urological Pathology grade group (ISUP GG) are well-established predictors of bone metastases and are used to stratify patients into risk groups. Appropriate selection of patients for bone imaging is essential, while unnecessary imaging should be avoided.

For many years, international guidelines have recommended bone imaging in patients with high-risk PC, as defined by the European Association of Urology (EAU), as well as in patients with unfavourable intermediate-risk disease [1]. The evidence base is largely derived from studies using [^99m^Tc]Tc-labelled diphosphonate planar bone scintigraphy.

Since then, more advanced imaging technologies, such as bone scintigraphy with SPECT/CT, [^18^F]sodium fluoride ([^18^F]NaF) PET/CT, and prostate-specific membrane antigen (PSMA) PET/CT, have been developed and are widely used in patients with newly diagnosed PC. These modalities have demonstrated superior diagnostic accuracy for the detection of bone metastases compared with planar bone scintigraphy [2–4]. The diagnostic pathway has also evolved. Although clinical T-staging according to the EAU guidelines is based on digital rectal examination, the introduction of MRI-targeted biopsies has led to risk-group migration, primarily through upgrading to higher ISUP GG categories [5].

Despite these advances, there is limited evidence regarding whether bone assessment remains warranted in the same patient populations originally identified using planar bone scintigraphy. Hence, current recommendations for bone assessment are still largely based on evidence obtained using planar bone scintigraphy, although risk classifications and imaging modalities have evolved [1, 6]. Furthermore, to our knowledge, no larger studies have systematically investigated the diagnostic accuracy and predictive factors for bone metastases on [^18^F]NaF PET/CT in newly diagnosed PC.

The aim of this study was (1) to assess the diagnostic accuracy of [^18^F]NaF PET/CT in a large contemporary cohort of patients with newly diagnosed PC and (2) to identify patient subgroups in whom [^18^F]NaF PET/CT for the detection of bone metastases may be redundant.

## Material and methods

### Participants

This retrospective, single-center study screened consecutive patients with newly diagnosed, biopsy-proven prostate adenocarcinoma who were referred from the Department of Urology for [^18^F]NaF PET/CT staging at Copenhagen University Hospital Herlev and Gentofte, a tertiary care center. The scans were performed between January 1, 2019, and December 31, 2020.

During the study period, patients were referred for bone imaging according to the EAU risk classification applicable at the time. This included patients with high-risk disease, including cT2c disease, and patients with intermediate-risk disease and ISUP GG 3. Patients with ISUP GG 2, PSA 10–20 ng/mL, and cT1–2b disease were classified as having favourable intermediate-risk disease and were therefore not routinely referred.

For the present study, the cohort was restricted to patients with high-risk disease according to the current EAU classification (cT3–4, ISUP GG 4–5, or PSA > 20 ng/mL) and patients with unfavourable intermediate-risk disease and ISUP GG 3 (cT1–2 and PSA ≤ 20 ng/mL).

From March 2019 onward, patients eligible for curative treatment underwent upfront MRI followed by systematic and MRI-targeted transrectal biopsies, whereas those unfit for curative treatment underwent standard ultrasound-guided systematic biopsies only.

Patients were excluded if they were diagnosed with a non-prostate malignancy, except for basal cell or squamous cell skin cancer, within 5 years before or up to 6 months after the [^18^F]NaF PET/CT.

### [^18^F]NaF PET/CT scan

A mean activity of 200 MBq (range, 165–349 MBq) was administered intravenously 30 min prior to image acquisition. The [^18^F]NaF PET was combined with either a low-dose CT or a diagnostic CT if soft-tissue evaluation was requested. Further details can be found in a previously published study [7].

### Image analysis and categorization

**Fig. 1:**
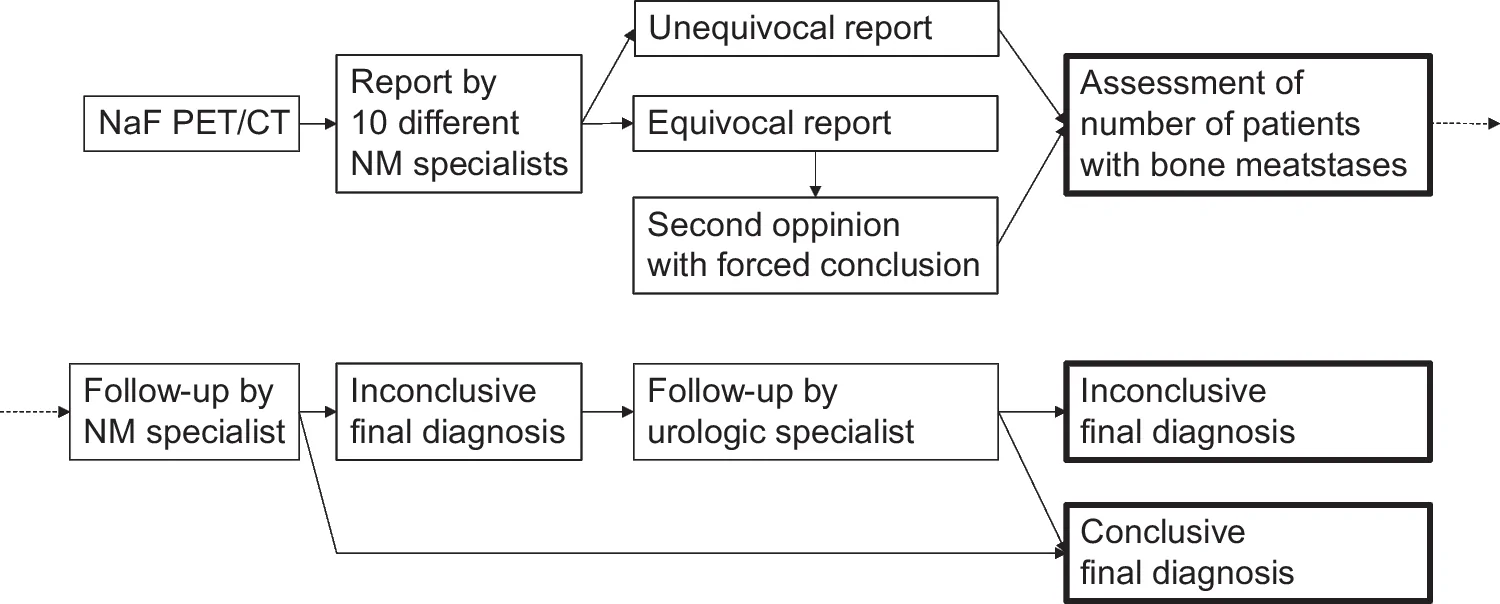
Process for assessing the number of patients with bone metastases and establishing the reference standard. NM = nuclear medicine

The [^18^F]NaF PET/CT scans were interpreted and reported by 10 nuclear medicine specialists, who collectively analyse more than 3,000 scans per year (Fig. 1). All scan reports were retrospectively reviewed by a nuclear medicine specialist from the study team and classified at the patient level as bone metastases, equivocal, or no bone metastases. For equivocal findings, two experts independently reanalysed the images and reached a final consensus diagnosis.

### Reference standard

A nuclear medicine specialist evaluated all available imaging obtained during the follow-up period in conjunction with clinical information from hospital records (Fig. 1). If the final diagnosis regarding bone metastases at the patient level remained inconclusive, a urologic specialist reviewed all available clinical data and determined the final diagnosis, which could still be inconclusive.

Factors supporting the presence of bone metastases, listed in descending order of evidential strength, included histologic verification, complementary MRI or PSMA PET/CT imaging demonstrating bone metastases, increasing fluoride uptake or newly emerging foci consistent with treatment failure, and a typical bone metastatic appearance of at least two foci on the index [^18^F]NaF PET/CT.

Factors arguing against the presence of bone metastases, listed in descending order of evidential strength, included an undetectable PSA after robot-assisted radical prostatectomy; complementary MRI or PSMA PET/CT imaging showing no evidence of bone metastases; absence of foci on follow-up imaging combined with a stable or decreasing PSA in the absence of systemic treatment; and consistently negative findings on follow-up imaging combined with a stable or decreasing PSA during ongoing systemic treatment.

### Statistics

Patients’ clinical characteristics are reported using descriptive statistics. Odds ratios (ORs) for the presence of bone metastases were estimated using logistic regression models. Univariate logistic regressions were performed separately for T-stage, PSA, and ISUP GG, and multivariate analyses were used to adjust for potential confounding among the three variables. ISUP GG 1 and 2 were pooled and used as reference category, since no bone metastases were observed in patients with ISUP GG 1. For each EAU risk group, sensitivity, specificity, positive predictive value, negative predictive value, and accuracy, along with 95% confidence intervals, were calculated. Statistical analyses were performed using RStudio version 4.4.0 (Posit Software).

## Results

A total of 773 patients underwent [^18^F]NaF PET/CT for primary staging. Of these, 29 patients were diagnosed with another cancer either before or shortly after the [^18^F]NaF PET/CT scan and were consequently excluded from the study. A further 10 patients with cT2c disease and ISUP GG 1–2 were excluded in accordance with the study population criteria described above. No bone metastases were detected in any of these 10 patients. Therefore, the final cohort eligible for further analysis comprised 734 patients (Table 1). Of these, 127 patients (17%) were classified as intermediate-risk with ISUP GG 3, and 607 patients (83%) were classified as high-risk.

**Table 1:** Demographic data of the study population stratified by EAU risk category

	Intermediate-risk with ISUP GG 3	High-risk	Total
Number of patients	127	607	734
Age (years), mean (2SD)	68 (55–81)	73 (57–89)	72 (56–88)
PSA (ng/mL), median (range)	9 (1.0–20)	36 (1.0–5677)	25 (1.0–5677)
PSA (ng/mL)			
< 10	68 (54%)	102 (17%)	170 (23%)
10–20	59 (46%)	95 (16%)	154 (21%)
> 20– < 50	n/a	180 (30%)	180 (25%)
50– < 100	n/a	84 (14%)	84 (11%)
≥ 100	n/a	146 (24%)	146 (20%)
Clinical T-stage			
T1	54 (43%)	43 (7%)	97 (13%)
T2	61 (48%)	102 (17%)	163 (22%)
T3	n/a	330 (54%)	330 (45%)
T4	n/a	101 (17%)	101 (14%)
Tx	12 (9%)	31 (5%)	43 (6%)
ISUP GG (*n*)			
1	n/a	6 (1%)	6 (1%)
2	n/a	78 (13%)	78 (11%)
3	127 (100%)	191 (31%)	318 (43%)
4	n/a	156 (26%)	156 (21%)
5	n/a	176 (29%)	176 (24%)

### Bone metastases on [^18^F]NaF PET/CT

The [^18^F]NaF PET/CT conclusion was equivocal in 24 of the 734 scans (3%). In these cases, two nuclear medicine specialists, blinded to the original clinical report, reviewed the [^18^F]NaF PET/CT scans together and reached a final, unequivocal consensus (Fig. 1). They determined that 20 of the 24 patients (83%) did not have bone metastases.

**Fig. 2:**
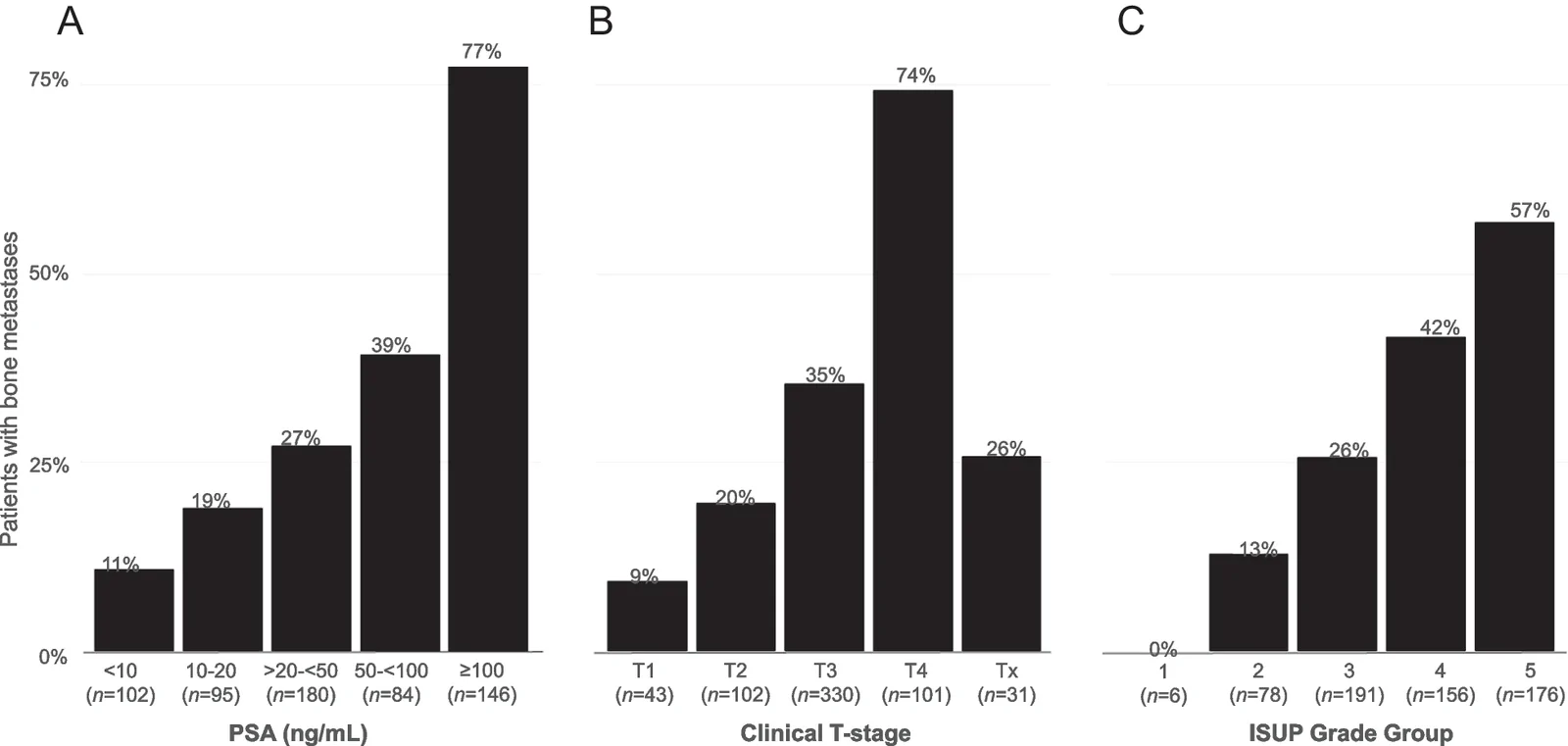
Prevalence of bone metastases on [^18^F]NaF PET/CT by PSA, clinical T-stage, and ISUP GG in patients with EAU high-risk PC

Of the 734 patients, 225 (31%) had bone metastases on [^18^F]NaF PET/CT (Table 2). Among high-risk patients, 224 (37%) had bone metastases, whereas only 1 patient (0.8%) in the intermediate-risk group with ISUP GG 3 had bone metastases on [^18^F]NaF PET/CT. In the high-risk group, there was a clear association between PSA, T-stage, and ISUP GG and the risk of detecting bone metastases (Fig. 2). This was confirmed in multivariate logistic regression analyses, with increasing ORs observed for higher PSA levels, T-stage, and ISUP GG categories (Table 3).

**Table 2:** Patients with bone metastases on [^18^F]NaF PET/CT

EAU risk group	Patients with bone metastases, *n* (proportion; 95% CI)	Patients with no bone metastases, *n* (proportion; 95% CI)	Total
Intermediate*	1 (0.8%; 0.02%–4.3%)	126 (99.2%; 95.7%–100%)	127
High	224 (37%; 33%–41%)	383 (63%; 59%–67%)	607
Total	225 (31%; 27%–34%)	509 (69%; 66%–73%)	734

^*^ Intermediate with ISUP GG 3

**Table 3:** Frequencies and odds ratios for bone metastases on [^18^F]NaF PET/CT in patients with EAU high-risk PC

	Proportion	Univariate OR (95% CI)	Multivariate OR (95% CI)
PSA (ng/mL)			
< 10	11/102 (11%)	Reference	Reference
10–20	18/95 (19%)	1.93 (0.86–4.34)	1.84 (0.80–4.25)
> 20– < 50	49/180 (27%)	3.09 (1.53–6.27)	3.29 (1.57–6.89)
50– < 100	33/84 (39%)	5.35 (2.49–11.49)	4.70 (2.10–10.51)
≥ 100	113/146 (77%)	28.33 (13.57–59.14)	20.36 (9.38–44.22)
Clinical T-stage			
T1	4/43 (9%)	Reference	Reference
T2	20/102 (20%)	2.38 (0.76–7.43)	1.85 (0.54–6.30)
T3	117/330 (35%)	5.36 (1.87–15.35)	3.80 (1.23–11.79)
T4	75/101 (74%)	28.12 (9.16–86.32)	8.69 (2.57–29.42)
Tx	8/31 (26%)	3.39 (0.92–12.52)	1.84 (0.43–7.77)
ISUP GG			
1–2	10/84 (12%)	Reference	Reference
3	49/191 (26%)	2.55 (1.22–5.33)	1.75 (0.77–3.99)
4	65/156 (42%)	5.29 (2.54–11.00)	3.31 (1.44–7.62)
5	100/176 (57%)	9.74 (4.72–20.10)	5.97 (2.63–13.55)

*OR* Odds ratio

### Follow-up

**Fig. 3:**
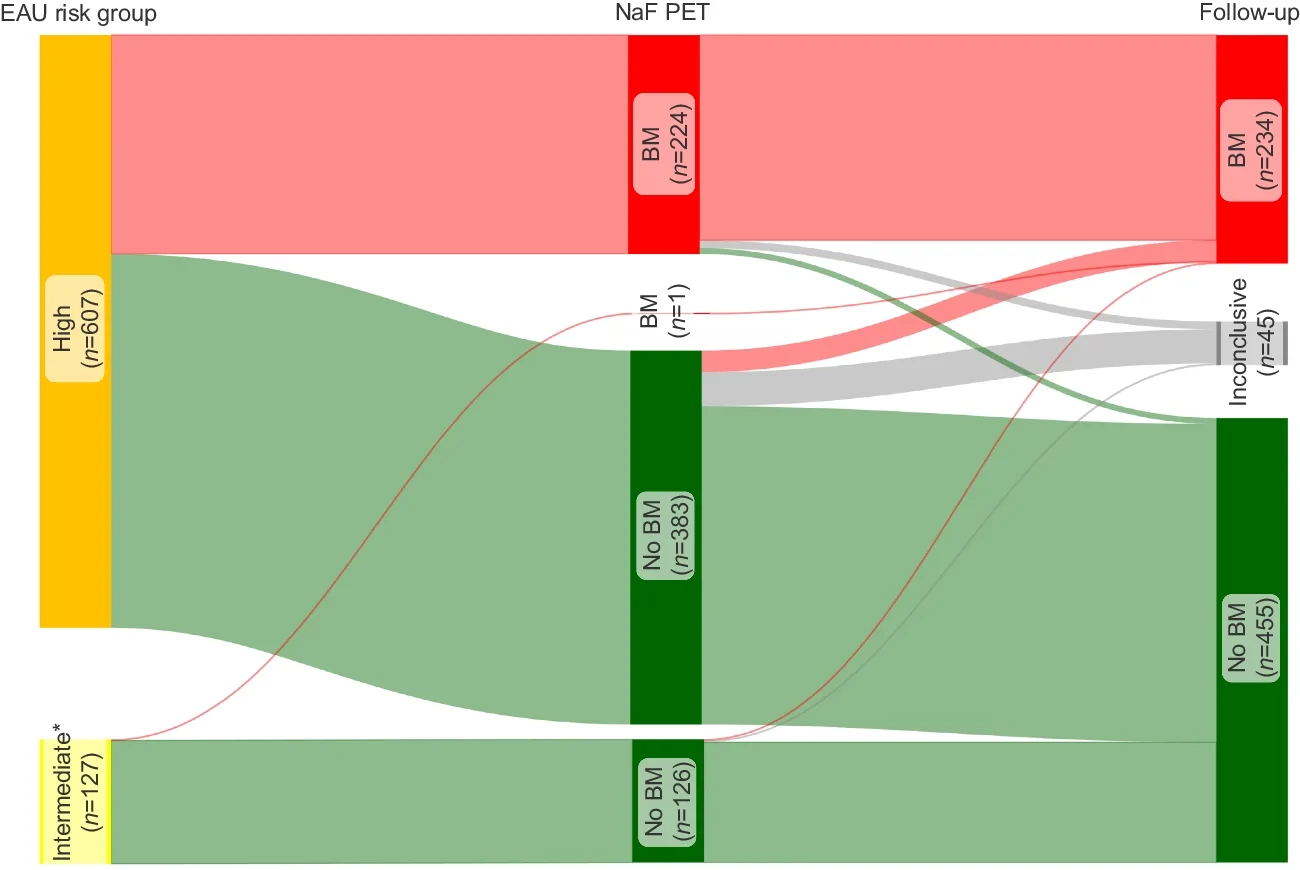
Sankey diagram illustrating patient flow from EAU risk group classification through [^18^F]NaF PET/CT findings (bone metastases present or absent) to final reference standard determination at follow-up. * Intermediate-risk with ISUP GG 3. BM = bone metastases

In 689 patients (94%), follow-up data were sufficient to establish a conclusive reference standard (Fig. 3). The sensitivity and specificity of [^18^F]NaF PET/CT for the detection of bone metastases in the cohort with a conclusive reference standard were 0.90 and 0.99, respectively (Table 4). Among high-risk patients, the corresponding values were 0.91 and 0.98.

**Table 4:** Diagnostic performance of [^18^F]NaF PET/CT for detection of bone metastases in the 689 patients with a conclusive reference standard

	Intermediate-risk with ISUP GG 3 (95% CI)	High-risk (95% CI)	Total (95% CI)
Sensitivity	0.50 (0.01–0.99)	0.91 (0.86–0.94)	0.90 (0.86–0.94)
Specificity	1.00 (0.97–1.00)	0.98 (0.96–0.99)	0.99 (0.97–1.00)
Accuracy	0.99 (0.96–1.00)	0.95 (0.93–0.97)	0.96 (0.94–0.97)
PPV	1.00 (0.02–1.00)	0.97 (0.94–0.99)	0.97 (0.94–0.99)
NPV	0.99 (0.96–1.00)	0.94 (0.91–0.96)	0.95 (0.93–0.97)
True-positive	1 (0.8%)	210 (37%)	211 (31%)
False-positive	0 (0.0%)	6 (1%)	6 (0.9%)
True-negative	123 (98%)	326 (58%)	449 (65%)
False-negative	1 (0.8%)	22 (4%)	23 (3%)

*PPV* Positive predictive value; *NPV* Negative predictive value

In the subgroup of 127 patients with unfavourable intermediate-risk disease and ISUP GG 3, only two patients were ultimately deemed to have bone metastases. One patient had a single sclerotic bone metastasis in the right pubic bone with avid [^18^F]NaF uptake. Subsequent [^68^Ga]Ga-PSMA-11 PET/CT confirmed the finding. The patient underwent radical prostatectomy as part of a clinical trial for oligometastatic disease. Histopathological examination of the prostatectomy specimen showed ISUP GG 5.

The second patient had no visible lesions on the [^18^F]NaF PET/CT at primary staging and underwent curatively intended external beam radiotherapy and 17 months of medical castration therapy, which was discontinued due to side effects. During follow-up, an [^18^F]NaF PET/CT performed 3½ years later revealed a suspicious lesion in the left pubic bone, and the index [^18^F]NaF PET/CT was classified as false-negative.

## Discussion

In this study, we investigated the risk of bone metastases detected by [^18^F]NaF PET/CT in 734 newly diagnosed PC patients with high-risk disease or intermediate-risk disease with ISUP GG 3. In the high-risk group, [^18^F]NaF PET/CT revealed bone metastases in 37% of patients. In contrast, among the 127 patients with intermediate-risk disease and ISUP GG 3, only 1 patient (0.8%) was found to have metastatic bone involvement. Follow-up suggested that one additional patient may have harboured bone metastases not detected by [^18^F]NaF PET/CT.

Nevertheless, the modality demonstrated high diagnostic performance, with a sensitivity of 0.90 and a specificity of 0.99 in the total cohort. The high diagnostic accuracy of [^18^F]NaF PET/CT for the detection of bone metastases is consistent with findings from previous studies, including those involving various oncologic diseases [2] and mixed PC populations [8], as well as a prospective study from our own group including only men with newly diagnosed PC [7]. To our knowledge, the present study is the largest assessing the diagnostic performance of [^18^F]NaF PET/CT in men with newly diagnosed PC.

The risk of bone metastases in patients with newly diagnosed PC is associated with biomarkers such as PSA, T-stage, and ISUP GG. However, the detection rate also depends on the imaging modality used. In nuclear medicine, three modalities are of particular interest: bone scintigraphy, [^18^F]NaF PET/CT, and PSMA PET/CT.

### Bone scintigraphy

The EAU guideline recommendations are based on studies in which planar bone scintigraphy is used [1]. In a single-center retrospective study of 853 consecutive patients across low-, intermediate-, and high-risk groups who underwent [^99m^Tc]Tc-MDP bone scintigraphy, Briganti et al. reported bone metastases in 18 patients (2%) and equivocal findings in 140 scans (16%) [9]. In comparison, only 24 of 734 scans (3%) were classified as equivocal in our study, consistent with previous findings for [^18^F]NaF PET/CT [2, 3]. The frequency of bone metastases in intermediate-risk patients with ISUP GG 3 cannot be directly derived from the study by Briganti et al. Nevertheless, their findings suggest that bone scintigraphy may be considered in patients with PSA > 10 ng/mL and cT2 disease, thereby including patients at intermediate risk. Our data support a more restrictive application of this recommendation, as [^18^F]NaF PET/CT identified bone metastases in only 1 of 127 intermediate-risk patients with ISUP GG 3.

Another retrospective study, conducted by Lin et al., included 703 patients with newly diagnosed PC who underwent whole-body [^99m^Tc]Tc-MDP bone scintigraphy [10]. In 298 cases (42%), the findings were equivocal, and these patients subsequently underwent additional imaging. The authors reported bone involvement in only 4% of patients with PSA < 20 ng/mL and ISUP GG < 4. Although T-stage was not included in their analyses, these findings are broadly consistent with our results.

A prospective, multicenter study by Zacho et al. included 635 patients who underwent whole-body planar [^99m^Tc]Tc-MDP bone scintigraphy [11]. No bone metastases were detected in a subcohort with PSA < 20 ng/mL, T-stage < T3, and ISUP GG < 4, findings that are consistent with the very low prevalence observed in our intermediate-risk cohort with ISUP GG 3. In line with other studies using bone scintigraphy, the number of equivocal scans was high (26%), although it decreased to 11% after additional imaging was performed.

The aforementioned studies were conducted before MRI-targeted biopsies became widely used in the diagnostic pathway. MRI-targeted biopsies may result in upgrading to higher ISUP GG categories and consequent risk-group migration [12]. Our cohort consisted of a mixed population in which some patients underwent MRI-targeted biopsies, whereas others underwent standard systematic biopsies. This change in biopsy practice may partly explain the low prevalence of bone metastases in patients with intermediate-risk disease and ISUP GG 3.

### [^18^F]NaF PET/CT

After intravenous administration, [^1^⁸F]fluoride is delivered to bone according to regional blood flow and exchanges with hydroxyl ions in hydroxyapatite to form fluoroapatite. Consequently, [^1^⁸F]NaF uptake reflects both bone perfusion and osteoblastic activity, which may be increased in bone metastases as well as in benign conditions [13]. Studies have demonstrated superior diagnostic accuracy of [^18^F]NaF PET/CT compared with bone scintigraphy for the detection of bone metastases [3, 14, 15], as well as high diagnostic accuracy for both [^18^F]NaF PET/CT and [^18^F]F-PSMA PET/CT-1007 [7]. Published data on the prevalence of bone metastases detected by [^18^F]NaF PET/CT are, to our knowledge, very limited. In a prospective study from our group including 160 patients with EAU high-risk disease, [^18^F]NaF PET/CT detected bone metastases in 25% of the cohort [7]. This prevalence was lower than the 37% observed in the present study, which could be partly explained by differences in patient selection between the two cohorts.

To our knowledge, the present study is the largest to assess the prevalence of bone metastases on [^18^F]NaF PET/CT in patients with newly diagnosed PC, encompassing 734 patients, and the first to evaluate the risk in a subpopulation with intermediate-risk disease and ISUP GG 3.

#### PSMA PET/CT

PSMA PET/CT has been implemented in many nuclear medicine departments and is valued for its superior sensitivity and specificity, particularly for the detection of lymph node metastases compared with CT [16–18], as well as for the detection of bone metastases compared with bone scintigraphy [4]. Some studies have examined risk factors associated with bone involvement on PSMA PET/CT, including a limited number focusing specifically on patients with intermediate-risk disease.

In a retrospective, multicenter study by Hagens et al., 396 patients with intermediate-risk disease and ISUP GG 3 underwent PET/CT using 4 different PSMA radioligands [19]. Bone metastases were detected in 14 patients (4%). Notably, 5 of 24 patients (21%) examined with [^18^F]F-PSMA-1007 were reported to have bone metastases. In comparison, bone metastases were detected in only 4 of the 145 patients (3%) examined using one of three [⁶⁸Ga]Ga-labelled PSMA radiopharmaceuticals, which is more consistent with the findings of our study. Indeed, [^18^F]F-PSMA-1007 is known to demonstrate uptake in non-specific bone lesions [20], and some false-positive findings may therefore have occurred, which may partly explain the higher detection rate observed with [^18^F]F-PSMA-1007.

Another retrospective study by Yaxley et al. included 1,253 consecutive patients with newly diagnosed PC across different risk groups, of whom 638 were classified as intermediate risk with ISUP GG 2 or 3 [21]. All patients underwent [⁶⁸Ga]Ga-PSMA-11 PET/CT for primary staging, which detected bone metastases in 18 of the 638 intermediate-risk patients (3%).

The above-mentioned studies are consistent with our [^18^F]NaF PET findings, although our intermediate-risk cohort did not include patients with ISUP GG 2, as these patients were not classified as unfavourable intermediate-risk at the time of inclusion. Nevertheless, bone metastases were detected in only 1 of 127 patients with intermediate-risk disease and ISUP GG 3. Given that ISUP GG 2 is associated with a lower risk profile than ISUP GG 3, the prevalence of bone metastases would be expected to be similarly low or lower in patients with ISUP GG 2.

The EAU guidelines recommend PSMA PET/CT or at least bone scintigraphy in patients with unfavourable intermediate-risk disease [1]. The National Comprehensive Cancer Network states that bone imaging, including PSMA PET/CT, [^18^F]NaF PET/CT, or bone scintigraphy, should be considered in this risk group [6]. Indeed, PSMA PET/CT is superior to CT for the detection of lymph node metastases [22, 23], and for detection of nodal involvement, PSMA PET/CT may be relevant in this subgroup. However, if a bone-remodelling imaging modality is used, i.e., [^18^F]NaF PET/CT or bone scintigraphy, our data indicate that bone metastases are rarely detected in patients with intermediate-risk disease and ISUP GG 3.

Furthermore, the extremely low prevalence of bone metastases in unfavourable intermediate-risk disease suggests that isolated PSMA-avid bone lesions should be interpreted cautiously in this subgroup. Given the low pretest probability of metastatic bone involvement, non-specific uptake may account for a substantial proportion of positive findings [20].

### Follow-up

In contrast to all previously discussed studies, our findings were validated against a reference standard. In the single patient with intermediate-risk disease and ISUP GG 3, in whom a bone metastasis was detected on [^18^F]NaF PET/CT, the finding was confirmed on a subsequent [^68^Ga]Ga-PSMA-11 PET/CT. In another intermediate-risk patient with ISUP GG 3, a follow-up [^18^F]NaF PET/CT performed 3½ years later revealed a pelvic bone metastasis, and the index [^18^F]NaF PET/CT was classified as false-negative. As illustrated by this case, the distinction between true-negative and false-negative findings may be challenging when follow-up serves as the reference standard, and some degree of misclassification cannot be excluded.

As indicated above, our study is not without limitations. The lack of histologic verification introduces a potential risk of misclassification in the reference standard. Moreover, systemic treatment initiated after the index scan may have masked bone metastases that would otherwise have become apparent during follow-up. Although [^18^F]NaF PET/CT detected bone metastases in only one patient with intermediate-risk disease and ISUP GG 3, additional false-negative findings cannot be entirely excluded.

In addition, prostate biopsy techniques were not uniform across the cohort. Some patients underwent systematic biopsies, whereas others underwent MRI-targeted biopsies. This heterogeneity may have influenced ISUP grading and, consequently, risk stratification.

Further, the very low prevalence of bone metastases in the intermediate-risk subgroup limits the evaluation of diagnostic performance. The sensitivity estimate was based on only two patients with bone metastases and was therefore highly imprecise.

Furthermore, the retrospective design introduces an inherent risk of selection bias and limits control over imaging indications and follow-up procedures. Finally, as this was a single-center study, the generalizability of the findings to institutions with different referral patterns or imaging protocols may be limited.

## Conclusion

[^18^F]NaF PET/CT demonstrated high diagnostic accuracy for the detection of bone metastases in the overall cohort. Bone metastases were detected only rarely in patients with intermediate-risk disease and ISUP GG 3. Routine use of [^18^F]NaF PET/CT in this specific subgroup therefore appears to have limited clinical value.

## Data Availability

The datasets generated during and/or analysed during the current study are available from the corresponding author on reasonable request.
